# Effect of Brief Mindfulness Induction on University Athletes’ Sleep Quality Following Night Training

**DOI:** 10.3389/fpsyg.2018.00508

**Published:** 2018-04-12

**Authors:** Chunxiao Li, Ying Hwa Kee, Lok Shan Lam

**Affiliations:** ^1^Department of Health and Physical Education, The Education University of Hong Kong, Ting Kok, Hong Kong; ^2^National Institute of Education, Nanyang Technological University, Singapore, Singapore

**Keywords:** mindfulness, night training, arousal, sleep disturbance, athletes

## Abstract

Given the need to alleviate sleep problems confronting athletes, the present experiment, conducted as much as possible in a naturalistic fashion that mimics daily life, seeks to examine whether a brief mindfulness induction immediately prior to sleep following night training can improve athletes’ sleep. A sample of university athletes (*n* = 80) was recruited and 63 of them were eligible to participate in this experiment. They were then randomly assigned into experimental group (*n* = 32) and control group (*n* = 31). Following night training and just prior to sleep, those in the experimental group received a self-administered brief 6-min mindfulness induction via a video clip, whereas the control group participants viewed a similar 6-min video devoid of mindfulness induction passively. Questionnaire-based measures of training intensity, pre-sleep arousal, state mindfulness, and sleep diary (i.e., level of rest, sleep duration, and overall sleep quality) were administered. Results showed that brief mindfulness induction reduced pre-sleep arousal, and improved level of rest and overall sleep quality, but not sleep duration. Pre-sleep arousal was also found to be a partial mediator in the relationship between the brief mindfulness induction and reported level of rest during sleep. These findings suggest that the brief mindfulness induction may be an effective approach for decreasing pre-sleep arousal and improving sleep quality after night training among athletes.

## Introduction

Ironically, sleep – a phase where one is seemingly doing nothing – is not necessarily easy to accomplish. The extensive research efforts placed on insomnia and other sleep disorders suggest that sleep is indeed not a straightforward affair (e.g., Broughton, 1968; Ohayon, 2002; Thorpy, 2017). For athletes in particular, sleep is essential, as it is an efficient way to promote post-training recovery (Halson, 2008). Although there are different modalities (e.g., cold-water immersion, psychological relaxation techniques, and sleep) for post-training recovery (Kellmann et al., 2018), considerable evidence has shown that sleep plays an active role in physiological and psychological processes such as removal of metabolic waste, prophylactic cellular maintenance, synaptic plasticity, emotional regulation, and memory functions (Fullagar et al., 2015). In this regard, sleep serves to facilitate both physiological and psychological restoration after training and competitions (Leeder et al., 2012), preparing the body for subsequent events (e.g., another bout of high-intensity training). In contrast, sleep disturbances can pose negative effects on athletes’ physical and psychological recovery. Sleep loss or continuous underrecovery has been related to numerous negative outcomes such as decreased performance during submaximal sustained exercise (Reilly and Edwards, 2007), diminished motor skill acquisition (Appleman et al., 2016), ill-being (Kellmann et al., 2018), low anaerobic power outputs (Souissi et al., 2008), overtraining syndromes (Jürimäe et al., 2004), and reduced cognitive functions (Fullagar et al., 2015), to name a few.

Despite the importance of sleep, studies suggest that athletes in general may not have sufficient sleep (e.g., Mah et al., 2011; Sargent et al., 2014). Furthermore, beyond the sleep duration deficiency, athletes had reported lower sleep quality (e.g., longer sleep latency, more awake time, and shorter sleep duration) than non-athletes in the same age and gender groups (Leeder et al., 2012). Given the need to alleviate the sleep deficiency condition confronting athletes, the present experiment, conducted as much as possible in a naturalistic fashion that mimics daily life, seeks to examine whether a brief mindfulness induction immediately prior to sleep post-night training can improve athletes’ sleep.

### Arousal and Sleep

Athletes’ sleep quality may be affected by a variety of factors such as nutrition statuses (Halson, 2014), religious activities (Herrera, 2012), training schedules (Sargent et al., 2014), training volumes (Li et al., 2017), and arousal levels (Kinsman et al., 2005). The issue of arousal, in particular, is a direct factor that can be dealt with through psychological strategies like mindfulness, and is therefore singled out for investigation here. To this end, arousal concerns the wide-ranging physiological and psychological activation of the body systems, which is important for regulating behavioral outcomes consciousness, information processing, and attention (Easterbrook, 1959). Early works, such as the one by Broughton (1968), had emphasized the need to examine the link between arousal and sleep. Other works also suggest that excessive arousal is likely to impair sleep quality (Nédélec et al., 2015). For example, training prior to sleep could heighten arousal and thereby affect sleep (Fullagar et al., 2015). These discussions illustrate the relevance of arousal regulation in relation to sleep issues.

Understandably, athletes’ arousal levels are elevated during physical training as their neural systems are stimulated by training stressors (Brisswalter et al., 2002). Further, their pre-sleep arousal is more likely to remain high if the compartment time between sleep and the completion of moderate intensity of training is less than 3 h (Higashiura et al., 2009). For athletes participating in high-intensity exercise, a compartment time of more than 3 h is needed to allow their arousal to return back to a lower level for sleep. Thus, the issue associated with arousal and sleep is particularly pertinent to how night training may affect sleep because in practice athletes’ bedtime is usually within 3 h of their night training (Robey et al., 2014). Here, if the compartment time is too short, night training may cause sleep problems due to high pre-sleep arousal level (Morin et al., 1999; Souissi et al., 2012). Effective interventions are therefore necessary to help athletes decrease arousal levels prior to sleep, and hopefully lead to alleviation of potential sleep problems.

The possibility of down regulating one’s arousal (relaxation) through mindfulness-related strategies (e.g., Ilan et al., 1985) led us to consider the applicability of brief mindfulness intervention (specifically focused attention toward one’s breathe) for sleep improvement. In particular, focusing attention to one’s breathe as a mindfulness practice has been shown to elicit lower heart rate and reported effort compared to loving kindness meditations and observing thoughts meditations (Lumma et al., 2015, 2017). Amihai and Kozhevnikov (2014) also reported that focused (Theravada) and distributed (Vipassana) attention meditations led to increased parasympathetic activation, which can inferred as lowered arousal states. Overall, paying attention to a predefined target continually as a practice seems to be useful for eliciting a relaxed state, and it is with this understanding that we set out to test the effects of attention-based mindfulness intervention on sleep in this study.

### Mindfulness and Sleep Quality

Mindfulness, as a strategy to reduce arousal before sleep, could be described as the psychological process of bringing attention to the present moment without judgment and interference (Brown and Ryan, 2003). As a psychological skill, mindfulness may entail refocusing one’s attention on a target, such as breathing or physical sensation, in a non-judgmental fashion repetitively for a prolonged period. Such a strategy potentially brings about relaxation, as suggested by Lumma et al. (2015), Amihai and Kozhevnikov (2014), and works on mindfulness-based stress reduction (MBSR; Chiesa and Serretti, 2009). Since psychosocial stress has been known to impair sleep (Akerstedt, 2006), it is not surprising that extant research suggests that mindfulness-related practice could be an effective approach for preventing and combating sleep-related problems (e.g., Shapiro et al., 2003; Carlson et al., 2004; Caldwell et al., 2010; Cincotta et al., 2011; Kanen et al., 2015). For example, an 8-week MBSR program attributed to Kabat-Zinn (1990), which involved weekly group sessions lasting 90 min each with a 3-h silent retreat on a Saturday facilitating participants onsite and home practice of mindful meditation, yoga, and sensitivity toward mind–body connection, was found to improve sleeping quality of cancer patients (Carlson et al., 2004). Cincotta et al. (2011) also found that the 8-week MBSR program decreased pre-sleep arousal and increased self-reported sleep quality among adults. Caldwell et al. (2010) found that increased mindfulness through movement-based mindfulness intervention accounted for changes in mood and perceived stress, and in part explained improved sleep quality. A recent meta-analysis also showed that mindfulness-based interventions can effectively decrease self-reported sleep disturbance among general populations (Kanen et al., 2015). Further, a systematic review on MBSR and sleep highlighted that improved sleep associated with mindfulness techniques may be linked to decreases in sleep-interfering cognitive processes such as worry (Winbush et al., 2007). In sum, research tends to suggest that participating in prolonged weeks of mindfulness-based intervention seems to benefit sleep, and favorable changes in perceived stress, worry, and mood seem to be associated with such interventions.

Although past research findings provided some evidence on the effectiveness of mindfulness-based intervention program on sleep, one may question whether these findings are generalizable for the athletic population given that athletes’ exertions, as a result of night training, were likely to be more vigorous leading up to their sleep. To this end, although elevated heart rate during sleep had been reported after vigorous activity and noted as a limitation in recovery during sleep, existing evidence were mixed regarding the impact of vigorous late night exercise on sleep (Myllymäki et al., 2011). Clearly, athletes’ sleep behavior is a complex matter, and could be different from non-athletes. Thus, the effects of mindfulness strategies for arousal reduction and better sleep in athletes may be different from non-athletes.

Majority of past investigations had focused on the effects of longer term mindfulness-based intervention on sleep (e.g., Shapiro et al., 2003; Carlson et al., 2004; Caldwell et al., 2010; Cincotta et al., 2011); thus, the adoption of brief mindfulness induction in this investigation is purposeful and fills a missing research gap. Clearly, there could be potential confounding factors arising from prolonged mindfulness interventions, such as improved mood (e.g., Caldwell et al., 2010), which makes it difficult for singling out mindfulness practice *per se* as the cause of improved sleep quality or quantity. Here, evidence of whether a brief bout of mindfulness practice affects sleep on the very same night reveals the effects of mindfulness on sleep more directly. Because intervention research on mindfulness strategies *per se* and sleep on athletes is nearly non-existent, the current intervention study would further supplement the extant literature on mindfulness and sleep.

Furthermore, some scholars highlighted the potential difficulty in getting athletes to use mindfulness strategies effectively (e.g., Nédélec et al., 2015). Meditation practice, for example, has been known to be challenging to women soccer players, particularly at the start of adopting such practice (Baltzell et al., 2014). Our efforts in testing the effects of a relatively simple mindfulness strategy over a short duration of a few minutes, immediately prior to sleep, hopefully contribute to the development of a simple strategy that can be readily applied by athletes without expectation for prolonged sitting meditation. Currently, research evidence suggests the effectiveness of brief mindfulness interventions (typically lasting for 5–10 min) in eliciting positive outcomes, such as empathic concern (Tan et al., 2014), stress-related blood pressure reactivity (Steffen and Larson, 2015), race discrimination (Lueke and Gibson, 2016), and compassion in medical decision making (Fernando et al., 2016). The effects of such brief interventions led us to consider whether a brief mindfulness induction would similarly improve athletes’ sleep.

### The Present Study

In summary, we argued that athletes’ sleep quality is likely to be affected by excessive arousal, particularly in the case of night training. As it is unclear whether a brief mindfulness intervention can decrease athletes’ pre-sleep arousal and thereby enhance athletes’ sleep quality, the present research aims to fill this research gap. A randomized controlled trial was conducted to examine the effectiveness of brief mindfulness induction on university athletes’ pre-sleep arousal and sleep quality after night training. According to the above literature review, it is hypothesized that our brief mindfulness induction could reduce pre-sleep arousal (Hypothesis 1; e.g., Campbell et al., 2015) and enhance sleep quality among athletes (Hypothesis 2; e.g., Kanen et al., 2015). It was also hypothesized that pre-sleep arousal will mediate the relationship between state mindfulness and sleep quality (Hypothesis 3; e.g., Cincotta et al., 2011).

## Materials and Methods

### Participants

University athletes (*n* = 80) who had regular training at night were invited to participate in this research. They were recruited from four sport teams (i.e., cross-country, handball, rugby, and volleyball) of a public university. Of those who were invited, 71 agreed to participate in this research. Eight participants who reported relatively high sleep quality compared with the rest were screened out (more details are introduced in due course) to avoid the ceiling effect. Finally, 63 participants (male = 42, female = 21, *M*_age_ = 21.16 years, *SD*_age_ = 1.79) constituted by 13 cross-country runners, 32 handball players, 5 rugby players, and 13 volleyball players remained in the study. All the participants were Chinese and were randomly assigned to the experimental group (*n* = 32; mindfulness induction) or the control group (*n* = 31) using a random number generator. There were no group differences in age, gender, and sport (*p* > 0.05). The participants received a cash coupon (approximately US$16) after completing the whole study.

### Measures

#### Sleep Quality

The 18-item Chinese version of the Pittsburgh Sleep Quality Index (PSQI; Buysse et al., 1989; Zhao et al., 2012) was used as a tool to screen for participants with relatively poor sleep quality over the last month. The PSQI assesses seven sleep components, including subjective sleep quality, sleep latency, sleep duration, habitual sleep efficiency, sleep disturbances, use of sleeping medication, and daytime dysfunction (Buysse et al., 1989). A global PSQI score which sums the scores of the seven components ranges from 0 to 21. A lower global score indicates better sleep quality, while participants with a global score greater than 5 is deemed as a poor sleeper (Buysse et al., 1989). The Cronbach’s α value of the scale was 0.67 in the current sample.

#### Training Intensity

The Rating Perceived Exertion scale (Borg, 1998) was used to measure participants’ training intensity immediately after night training. Participants were asked to rate how hard they felt like their body was working in terms of heart rate, respiration rate, sweating, and muscle fatigue. They rated on a scale ranging from 6 (no exertion at all) to 20 (maximal exertion). Rating scores lower than 10, between 11 and 14, and higher than 15 indicate low, moderate, and high training intensities, respectively (Borg, 1998).

#### Arousal Level

The 16-item Chinese version of the Pre-sleep Arousal Scale (PASA; Nicassio et al., 1985; Chen et al., 2011) was used to measure participants’ pre-sleep arousal level. Participants were required to describe how intensely they generally experienced each symptom (e.g., “Worry about falling asleep”) as they attempted to sleep using a five-point Likert scale ranging from 1 (not at all) to 5 (extremely). The total scale score ranges from 16 to 80. A higher score indicates a higher pre-sleep arousal level (Nicassio et al., 1985). The Cronbach’s α values of this scale for data collected right after night training (Time 1), right before the brief mindfulness induction (Time 2), and immediately after the induction (Time 3) were 0.92, 0.86, and 0.88, respectively.

#### State Mindfulness

The five-item Chinese-translated State Version of the Mindful Attention Awareness Scale (Brown and Ryan, 2003; Chang et al., 2011) was used to measure participants’ state mindfulness as a manipulation check immediately post-intervention. The items (e.g., “I was finding it difficult to stay focused on what was happening”) were measured using a seven-point Likert scale, anchored with 0 (not at all) and 6 (very much). The item scores were reversely coded and averaged. A higher scale score refers to a greater state mindfulness level. In the current sample, the Cronbach’s α value of the scale was 0.81.

#### Sleep Diary

To assess variables of interest concerning sleep, three items were adapted from the Sleep and Health Research Laboratory’s Sleep Diary (Sethi, 2012) to measure participants’ level of rest, sleep duration, and overall sleep quality. The instrument adopts a 10-point Likert scale for assessing level of rest and overall sleep quality, and was filled immediately when participants woke up in the morning. Participants were asked to report how they felt (1 = “very tired,” 10 = “rested/alert”) as an indication of level of rest, as well as perceived overall sleep quality through the question “Overall, what was the quality of your sleep last night?” (1 = “light,” 10 = “deep”). They also gave an estimate for their total sleep duration last night. High correlations between the sleep diary and PSQI scores were found previously (Sethi, 2012).

### Procedure

The study was conducted in accordance with the Declaration of Helsinki and with the approval of the Human Research Ethics Committee of the Education University of Hong Kong. Head coaches of four sport teams were approached to invite their athletes to participate in this research. Seventy-one out of eighty participants agreed to participate in this research and signed informed consent forms. Eight participants who reported relatively high sleep quality (PSQI < 4) were screened out to avoid the ceiling effect. The remaining participants (*n* = 63) had a mean PSQI score of 6.81 (*SD* = 2.28).

**FIGURE 1:**
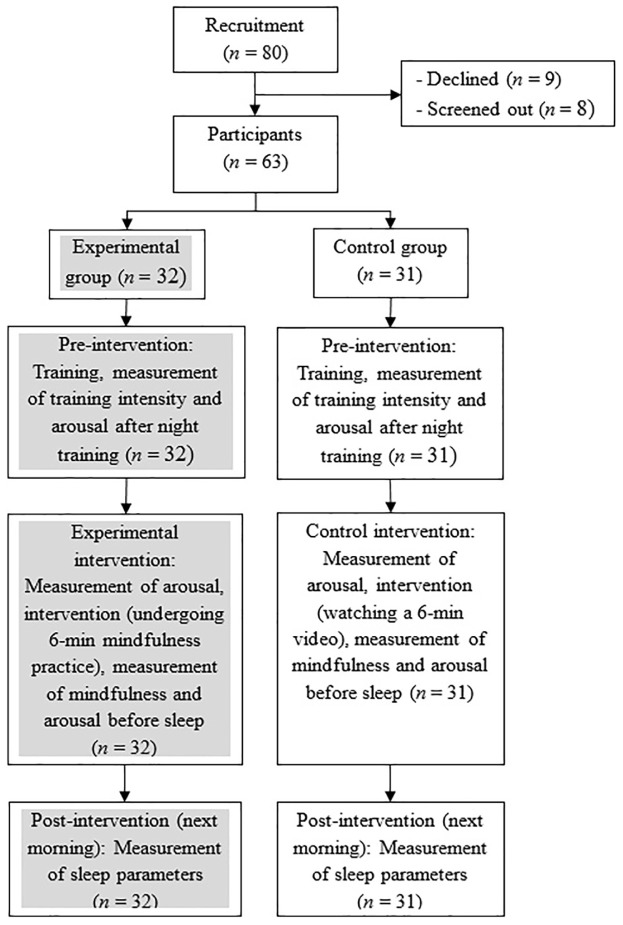
Study procedure and disposition of participants.

This study involved participants receiving face-to-face instructions from the researchers, as well as carrying out part of the experiment protocol away from the researchers independently. **Figure 1** shows the procedure of this experiment. The experiment was carried out 1 week after screening out participants with relatively good sleep quality. The remaining participants were briefed that the purpose of the study was to examine whether listening to music before sleep can enhance their sport performance. This deception is necessary to prevent threat of internal validity related to expectancy. As this is a study that requires participants to carry out specific tasks on their own surrounding their sleep time, they were given a checklist of the tasks to follow when they were briefed beforehand. They were also provided with a manipulation video file for subsequent use. Any doubts were cleared before they proceeded with the study.

During the course of experiment, participants first filled up the questionnaires pertaining to their exercise intensity and arousal level immediately after one of their night training sessions, which spanned from 7 p.m. to 10 p.m. All the survey forms except for the aforementioned questionnaires were filled out by the participants without research assistants’ supervision during the experiment. Later in the same night in participant’s own bedroom, just before they were ready to sleep, they rated their arousal level before undergoing the brief mindfulness induction. To receive the induction, the participants played the assigned 6-min video file through their smart phone to watch the video with headsets on. All participants were instructed through the video to place their left index finger under the nostril while breathing normally. The experimental group members were specifically instructed in the video to pay attention to the sensation of the index finger when they breathed in and out. Bell chimes were also sounded periodically to serve as reminder for them to maintain mindful attention. The control group’s video file did not feature the mindful attention instructions but they could hear the same audio bell chimes. As the scope of this research focuses on brief mindfulness induction (typically lasting for 5–10 min; e.g., Fernando et al., 2016; Lueke and Gibson, 2016), the 6-min induction was purposefully adapted from Kee et al. (2013), previously found to significantly increase participant’s state mindfulness level relative to that of the control group. Once the video ended, the participants reported their state mindfulness (as manipulation check) and arousal levels, and then proceeded to sleep. In the subsequent morning, the participants completed the modified sleep diary immediately upon waking. Finally, the participants returned their completed checklist to the researchers within 1 week. All participants reported that they followed the whole experiment procedure without deviation. The participants received the cash coupon and were debriefed.

### Data Analysis

Descriptive statistics including means and standard deviations were used to describe the study outcomes, including perceived training intensity, pre-sleep arousal, state mindfulness level, and sleep parameters (i.e., level of rest, sleep duration, and overall sleep quality). Independent *t*-tests were used to compare the group difference on training intensity and state mindfulness.

To test Hypothesis 1 (brief mindfulness induction could reduce pre-sleep arousal), a 3 (Time) × 2 (Group) repeated measures analysis of variance was adopted to examine the group difference on arousal level across the three measurement points (Time 1–Time 3). Follow-up simple effect tests with Bonferroni corrections (0.05/3) were conducted to compare group differences if there was a significant effect. To test Hypothesis 2 (brief mindfulness induction could enhance sleep quality among athletes), independent *t*-tests were used to compare the group differences on the sleep parameters. The magnitude of group difference was measured based on Cohen’s *d* effect size. According to Cohen (1992), *d* values of 0.2, 0.5, and 0.8 present a small, moderate, and large effect, respectively. To investigate Hypothesis 3 (pre-sleep arousal will mediate the relationship between mindfulness induction and sleep quality), mediation analyses with bootstrapping were used to examine whether pre-sleep arousal is a significant mediator in the relationship between the brief mindfulness induction (group was entered as independent variable) and each of the sleep parameters. In this study, the difference between arousal measure immediately before and after the induction (Time 2–Time 3) is used as the measure of pre-sleep arousal to account for influence of initial difference in the mediation analyses (Holmes, 1984). We used 5,000 bootstrap samples to generate bias-correct confidence interval (CI) and a 95%CI that does not include zero indicates a significant indirect effect (Hayes, 2013). All the analyses were performed with SPSS 22.0 and the significant level was set at 0.05.

## Results

### Descriptive Statistics: Training Intensity and Manipulation Check

Both groups reported a moderate to high training intensity (Borg, 1998; see **Table 1**). The results of the independent sample *t*-test showed that there was no significant group difference on training intensity (*t*[61] = -0.94, *p* = 0.35, *d* = -0.23). The experimental group reported a significantly higher state mindfulness level than the control group (*t*[61] = 2.74, *p* = 0.008, *d* = 0.69), suggesting the usefulness of the 6-min mindfulness induction in increasing experimental group participants’ state mindfulness level.

**Table 1:** Group comparisons of training intensity, state mindfulness, pre-sleep arousal, and sleep parameters.

	Range	Experimental group	Control group	*p*-value
				
		*M* (*SD*)	*M* (*SD*)	
The intensity of night training	6–20	14.16 (2.70)	14.77 (2.53)	0.35
State mindfulness	0–6	3.84 (1.03)	3.17 (0.91)	0.008
Time 1 arousal level	16–80	42.00 (14.03)	42.26 (10.40)	0.93
Time 2 arousal level	16–80	31.69 (9.22)	33.13 (6.53)	0.48
Time 3 arousal level	16–80	25.56 (6.38)	31.10 (7.13)	0.002
Level of rest	1–10	6.22 (1.50)	5.03 (1.62)	0.004
Sleep duration (hours)	2–11	7.66 (0.94)	7.13 (1.87)	0.16
Overall sleep quality	1–10	6.94 (1.39)	5.65 (1.87)	0.003


*Time 1 = immediately after night training; Time 2 = right before the brief mindfulness induction; and Time 3 = immediately after the induction.*

### Hypothesis 1: Pre-sleep Arousal

The results of repeated measures analysis of variance indicated that there was a time effect on arousal (*F*[2,61] = 94.17, *p* < 0.001) and the participants’ arousal level decreased along time (see **Table 1**). The group effect was not significant (*F*[2,61] = 1.40, *p* = 0.24). Further, a time and group interaction on arousal level was detected (*F*[2,61] = 3.59, *p* = 0.03). Based on the results from the simple effect tests, the two groups did not differ significantly in terms of arousal level at Time 1 (*t*[61] = -0.08, *p* = 0.93, *d* = 0.02) and Time 2 (*t*[61] = -0.71, *p* = 0.48, *d* = 0.18). However, the arousal level at Time 3 of the experimental group was significantly lower than the control group (*t*[61] = -3.24, *p* = 0.002, *d* = 0.82). Thus, Hypothesis 1 was confirmed.

### Hypothesis 2: Sleep Quality

The results of the independent sample *t*-test showed that the experimental group had a higher level of rest (*t*[61] = 3.02, *p* = 0.004, *d* = 0.76) and overall sleep quality (*t*[61] = 3.12, *p* = 0.003, *d* = 0.78) than the control group. Although the experimental group tended to report longer sleep duration (*M* = 7.66, *SD* = 0.94) than the control group (*M* = 7.13, *SD* = 1.87), there was no significant group difference (*t*[43.83] = 1.44, *p* = 0.16, *d* = 0.36). Consequently, Hypothesis 2 was partially supported.

### Hypothesis 3: Indirect Effects

**FIGURE 2:**
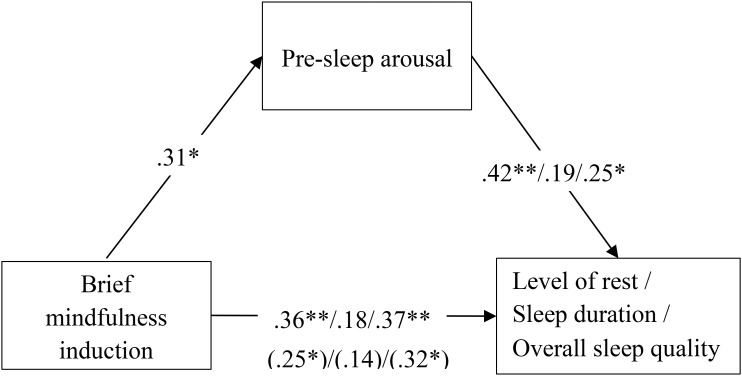
Standardized regression coefficients for the direct relationship between brief mindfulness and sleep parameters as mediated by pre-sleep arousal. The indirect standardized regression coefficients are represented in parentheses. ^∗∗^*p* < 0.01; ^∗^*p* < 0.05.

**Figure 2** presents the standardized regression coefficients among mindfulness, pre-sleep arousal, and sleep parameters. The results of mediation analysis with bootstrapping indicated that the brief mindfulness induction positively predicted pre-sleep arousal (*B* = 0.83, *SE* = 0.39, 95%CI [0.05, 1.61]), level of rest (*B* = 4.09, *SE* = 1.58, 95%CI [0.92, 7.26]), and overall sleep quality (*B* = 1.13, *SE* = 0.43, 95%CI [0.26, 2.00]), but not sleep duration (*B* = 0.40, *SE* = 0.39, 95%CI [-0.38, 1.18]). Moreover, there was a significant indirect effect of the brief mindfulness induction on level of rest (*B* = 0.35, *SE* = 0.19, 95%CI [0.06, 0.83]). However, the indirect effects of the brief mindfulness induction on sleep duration (*B* = 0.14, *SE* = 0.16, 95%CI [-0.05, 0.64]) and overall sleep quality (*B* = 0.16, *SE* = 0.16, 95%CI [-0.07, 0.58) were not significant. These results suggest that the pre-sleep arousal was a partial mediator in the relationship between the brief mindfulness induction and level of rest. Pre-sleep arousal did not mediate the relationship between the brief mindfulness induction and sleep duration/overall sleep quality. Therefore, Hypothesis 3 was partially supported.

## Discussion

The current research examined the effects of brief mindfulness induction on university athletes’ pre-sleep arousal and sleep quality after moderate to high-intensity night training. It was hypothesized that our brief mindfulness induction could reduce pre-sleep arousal (Hypothesis 1) and enhance sleep quality among athletes (Hypothesis 2). It was also hypothesized that pre-sleep arousal will mediate the relationship between mindfulness induction and sleep quality (Hypothesis 3). In summary, our results suggest that the brief mindfulness induction reduces pre-sleep arousal and improves sleep quality in terms of level of rest and overall sleep quality, but not duration. Pre-sleep arousal is also found to be a partial mediator in the relationship between the brief mindfulness induction and reported level of rest during sleep.

Before discussing the various findings, it must be noted that the 6-min brief mindfulness induction was delivered by way of a video clip and self-administered by participants prior to sleep. Results of the manipulation check suggest that it increases the experimental group members’ state mindfulness relative to that of the control, which is consistent with past research (Kee et al., 2013). Essentially, the mindfulness induction directs participants’ attention toward their own breath by prompting them to feel the sensation of air on their index finger placed under their nostril continuously for 6 min. In comparison with typical breathing meditation which involves sitting quietly while paying attention to the sensation of air at the nostril (e.g., Barnhofer et al., 2010), this abridged version of mindfulness induction makes use of intrinsic feedback from the sensation of one’s finger to facilitate participants’ mindful effort toward their breathes. Given that no prior training on mindfulness was provided to the participants, the overall results are encouraging, perhaps signaling that such a manipulation can be easily adopted by athletes, at least for the purpose of promoting sleep.

Given the utility of the mindfulness intervention in eliciting state mindfulness, the outcome of Hypothesis 1 is not surprising as lower arousal had been associated with mindfulness practice (Chiesa and Serretti, 2009). Previously, Cincotta et al. (2011) also found that an 8-week mindfulness practice decreased adults’ pre-sleep arousal. While the time scales are different, the finding about mindfulness lowering pre-sleep arousal is consistent. Nonetheless, the special interest in this study is that of the utility of such a strategy on athletes’ sleep and arousal. Our participants reported moderate to high training intensities immediately after the night training (Borg, 1998) as the training stressors stimulated participants’ neural system and elevated their arousal (Brisswalter et al., 2002). In general, participants’ arousal decreased along the three measurement points during our study. However, a moderate arousal level at Time 2 (right before the brief mindfulness induction) is noted. This heightened awareness could cause sleep problems if left unchecked (Nicassio et al., 1985).

Following the induction phase, the experimental group reported a significantly lower arousal level than the control group, with a large effect size. The mindfulness induction allowed experimental group members to focus on the present moment through attention to their breaths. As attention is temporarily directed to a neutral sensation, participants’ habitual attention to thoughts of the past and future, such as mistakes made in training or worry about impending competitions, may have been suspended. Further, Mikulas (2011) articulated that concentration form of meditation (e.g., the current intervention) has the potential to bring about calmness and relaxation in one’s mind and biological relaxation response in the body (Benson and Klipper, 1992). For those participants who followed the mindfulness induction, they demonstrated that they had benefited from it by lowering arousal at least immediately after it.

In addressing Hypothesis 2, results show that the experimental group reported a higher level of rest and overall sleep quality than the control group as a result of the induction. This finding concurs with previous studies where regular mindfulness practice was found to be effective for enhancing sleep quality among general populations (e.g., Winbush et al., 2007; Howell et al., 2008; Caldwell et al., 2010; Hülsheger et al., 2015). We extend the current understanding by demonstrating that a short bout of mindfulness practice prior to sleep could lead to self-reported higher level of rest and overall sleep quality. The positive impact of such a pre-sleep mental practice is welcomed as both sleep quality and quantity are important for athlete recovery (Halson, 2008). Although the current study revealed that the mindfulness induction did not increase total sleep duration, it could be a case of a ceiling effect as sleep duration of our participants were between 7 and 8 h, which meets the requirement of “healthy” night sleep (Krystal and Edinger, 2008). If the participants in this study had a total sleep duration of less than 7 h, the results may be different as there might be more room for increasing their sleep duration. Nevertheless, the overall higher sleep quality resulting from the brief mindfulness induction underpins the potential for such mindfulness-based pre-sleep mental practice to be further utilized and researched upon.

The most important finding arising from this study would be the significant partial mediation through pre-sleep arousal observed within the mindfulness induction and level of rest relationship. Plainly put, pre-sleep arousal is a possible mechanism by which our brief mindfulness induction brought about, leading to reported greater level of rest during sleep. This finding is encouraging as it demonstrates that athletes’ pre-sleep arousal and level of rest can be enhanced even with a short bout of mindfulness induction, experiencing such an efficacious effect may encourage athletes to consider undergoing a longer term intervention (e.g., 8-week MBSR) to reap the other benefits of mindfulness for sport performance (e.g., Bernier et al., 2009). As previously alluded, the link between mindfulness practice and lowered arousal is well established from the perspectives of biological relaxation response (Benson and Klipper, 1992) and in MBSR work (e.g., Cincotta et al., 2011). Corroborating the current findings with previous works, we have greater confidence to speculate that a brief bout of mindfulness practice prior to sleep could lower arousal level, setting the conditions for lower likelihood of sleep-interfering cognitive processes (Winbush et al., 2007), thereby leading to better sleep among athletes. In a way, since sleep is essentially a state where “no activity” is pursued, lowering one’s arousal prior to sleep facilities non-striving and letting go of any concerns bothering the individual for the day. As acute effects of mindfulness are not usually studied in research featuring a prolonged program like MBSR, the current study which focuses on acute effects of mindfulness practice yields findings that contribute to overall understanding of the issue.

Given that the mediation effects of pre-sleep arousal are only partial, it is worthwhile to discuss other related factors. A previous cross-sectional questionnaire-based study examining possible mediators between mindfulness and sleep by Campbell et al. (2015) found basic psychological needs satisfaction composite (autonomy, competence and relatedness) to potentially represent a critical explanatory mechanism. In explaining why needs satisfaction might be important, Campbell et al. (2015) suggest:

“The open awareness characteristic of mindfulness likely facilitates attention to one’s internal world and psychological functioning and in doing so, increases the likelihood that one will act in ways that fulfill basic psychological needs, which in turn enables better sleep outcomes. In addition, when encountering problems with falling asleep, mindful individuals may be more able to accept sleep-interfering thoughts rather than resist them, which would be further conducive to their sleep.” (p. 203)

While Campbell et al. (2015) based their rationalization on data gathered at the dispositional level (particularly in terms of basic psychological needs’ satisfaction), they provided some support regarding the usefulness of mindfulness-associated qualities in promoting sleep. We would further add that a well-intended short bout of mindfulness practice, whist bringing about lower arousal and relaxation, could also facilitate one’s attention toward his/her internal world and psychological functioning. The notion of bare awareness, fundamental to mindfulness, undergirds this (Shapiro et al., 2006). To this end, the second component of mindfulness expounded by Bishop et al. (2004), which pertains to adopting an orientation characterized by curiosity, openness, and acceptance, may have relevance.

Further, while improved overall sleep quality was observed among the experimental group participants, pre-sleep arousal was not a mediator in the relationship between the brief mindfulness induction and overall sleep quality. One possible explanation could be that the item measuring overall sleep quality is more obscure and less sensitive than the item measuring level of rest. When the participants were instructed to rate their overall sleep quality, they may evaluate a lot of components such as sleep latency, sleep duration, and sleep disturbances. In comparison, it was very straightforward for the participants to answer the question about their level of rest. Another possibility is that the underlying mechanism between the brief mindfulness induction and overall sleep quality may be explained by other factors rather than arousal. For example, the relationship between the mindfulness practice and sleep quality was found to be partially accounted by the changes in mood and perceived stress (Caldwell et al., 2010). Another candidate for mediation worth examining would be basic psychological needs satisfaction (Campbell et al., 2015).

### Limitations and Implications

We acknowledge that the current study presents several limitations despite the robust statistical findings observed. First, although a checklist was used to assess the intervention fidelity, there is always likelihood that some participants may not have given honest responses. However, it is worthy to note that our experiment was conducted in a natural setting rather than in a well-controlled environment, an essential feature of sleep intervention study which increases the external validity. Second, participants of the study were university athletes from four sports teams after moderate- to high-intensity night training; thus, the study findings may not be generalized into other groups under different training intensity. As training intensity or fatigue induced by training has been found to affect athletes’ sleep (Buchheit et al., 2016; Li et al., 2017), future studies can target various populations such as elite athletes including those with severe sleep problems under varying training intensity to see whether the current study findings can be replicated. Third, gender effects were not extensively evaluated in this study, and would be worthy of further investigation as menstrual cycle has been known to be affect sleep (Baker and Driver, 2007) while mindfulness has been proposed as a possible remedial strategy in this regard (Lustyk et al., 2011). Finally, self-reported measures were used to measure sleep parameters, and future research should use objective assessments of sleep such as through tri-axial accelerometer (Nam et al., 2016).

Despite the limitations, encouraging results were found in the current research. From a practical perspective, given its ease in implementation, a 6-min brief-mindfulness induction can be used as an effective and economic way to enhance athletes’ level of rest and overall sleep quality after moderate to high-intensity night training. Given the advancement in information technology, there exists a plethora of mobile applications aimed at guiding mindfulness practice that can be used for achieving better sleep (e.g., Headspace, see^1^). Further studies may also examine the dose–response effect between the duration of similar brief-mindfulness induction and sleep quality. Our study findings provide relevant effect sizes for estimating the power and sample size in future research.

## Conclusion

The brief mindfulness induction used in the current study is effective in reducing pre-sleep arousal level, which may contribute to the enhanced level of rest and overall sleep quality among university athletes after night training. It is hoped that this research will proliferate the research and practice of using brief mindfulness induction to enhance sleep quality, particularly that of athletes. Putting this research in the context of a bigger picture, mindfulness strategies as a skill can be thought of as an individualized recovery strategy that facilitates athletes’ recovery, and is aligned with the recommendations in the recent consensus statement on Recovery and Performance in Sport (Kellmann et al., 2018).

## Author Contributions

CL and LL conceived the overall study design and conducted the data collection. CL and YK analyzed the data. All the authors contributed to the manuscript writing.

## Conflict of Interest Statement

The authors declare that the research was conducted in the absence of any commercial or financial relationships that could be construed as a potential conflict of interest.

## Footnotes

1 https://www.headspace.com/
